# Graph-Based Self-Training for Semi-Supervised Deep Similarity Learning

**DOI:** 10.3390/s23083944

**Published:** 2023-04-13

**Authors:** Yifan Wang, Yan Huang, Qicong Wang, Chong Zhao, Zhenchang Zhang, Jian Chen

**Affiliations:** 1 Department of Computer Science and Technology, School of Information, Xiamen University, Xiamen 361005, China; 2 Shenzhen Research Institute, Xiamen University, Shenzhen 518000, China; 3 College of Computer and Information Sciences, Fujian Agriculture and Forestry University, Fuzhou 350002, China; 4 Third Institute of Oceanography, MNR, No. 178 Daxue Road, Xiamen 361005, China

**Keywords:** semi-supervised learning, self-training, graph structural learning

## Abstract

Semi-supervised learning is a learning pattern that can utilize labeled data and unlabeled data to train deep neural networks. In semi-supervised learning methods, self-training-based methods do not depend on a data augmentation strategy and have better generalization ability. However, their performance is limited by the accuracy of predicted pseudo-labels. In this paper, we propose to reduce the noise in the pseudo-labels from two aspects: the accuracy of predictions and the confidence of the predictions. For the first aspect, we propose a similarity graph structure learning (SGSL) model that considers the correlation between unlabeled and labeled samples, which facilitates the learning of more discriminative features and, thus, obtains more accurate predictions. For the second aspect, we propose an uncertainty-based graph convolutional network (UGCN), which can aggregate similar features based on the learned graph structure in the training phase, making the features more discriminative. It can also output the uncertainty of predictions in the pseudo-label generation phase, generating pseudo-labels only for unlabeled samples with low uncertainty; thus, reducing the noise in the pseudo-labels. Further, a positive and negative self-training framework is proposed, which combines the proposed SGSL model and UGCN into the self-training framework for end-to-end training. In addition, in order to introduce more supervised signals in the self-training process, negative pseudo-labels are generated for unlabeled samples with low prediction confidence, and then the positive and negative pseudo-labeled samples are trained together with a small number of labeled samples to improve the performance of semi-supervised learning. The code is available upon request.

## 1. Introduction

Semi-supervised learning is a schema for network training using a small amount of labeled data and a large amount of unlabeled data. The current semi-supervised learning methods are mainly categorized into consistency regularization methods [1,2] and pseudo-labeling methods [3,4]. Consistent regularization methods aim to keep the outputs of the model constant under perturbations. For example, Sajjadi et al. [5] proposed the π model. It conducts two separate data augmentations for inputs and predicts the augmented inputs separately using a deep network, then minimizes the distance between the two predictions by a consistency loss function. However, the consistency regularization methods mostly rely on data augmentation strategies, thus their generalization ability is limited.

In contrast, pseudo-labeling methods are independent from data augmentations. They aim to generate pseudo-labels for unlabeled data and then train the network along with a small amount of labeled data. In pseudo-labeling methods, self-training methods [6,7] are the most widely studied methods, and such methods have three steps. Firstly, the network is pre-trained with a small amount of labeled data. Secondly, the pre-trained network is used to generate pseudo-labels by classifying and predicting unlabeled data. Finally, the network is trained with pseudo-labeled data and small amounts of labeled data. However, the accuracy of the pseudo-labels limits the performance of the pseudo-labeling-based methods. Specifically, pseudo-labels are mostly obtained from the predictions of the model, which is not always reliable. In addition, if the model achieves high confidence on wrong predictions, the model will continue to learn incorrectly.

It is worth mentioning that Rizve et al. [8] proposed to use uncertainty to determine whether predictions of the network are reliable and to generate pseudo-labels on low confidence to enrich supervised signals. However, this method does not take into account the correlation between labeled and unlabeled samples. In fact, self-training methods are often trained based on the assumption that results with high confidence tend to be correct. However, this assumption may only hold if the features are discriminative in the data space. It is well known that the features of a small amount of labeled data are generally discriminative after supervised training of the network. If the correlation between unlabeled data and labeled data can be further considered so that unlabeled data are close to similar labeled data, the self-training model will be more accurate in predicting unlabeled data and generating more accurate pseudo-labels, which will be beneficial to the network’s self-training. Therefore, it is necessary to consider the potential similarity relationship between unlabeled and labeled data.

The key to improving the performance of self-training methods lies in two aspects: learning more discriminative features and generating more accurate pseudo-labels. To this end, we propose a positive and negative self-training framework based on graph-based deep uncertainty, which consists of two key models: the similarity graph structural learning (SGSL) model and the uncertainty-based graph convolutional network (UGCN). The proposed self-training framework consists of three stages. In the first stage, the entire network is trained in a supervised manner using a small amount of labeled data. In the second stage, the network model is adjusted to the test mode and the unlabeled data are fed into the network for classification prediction. The high and low confidences are filtered to generate pseudo-labels; the pseudo-labels include positive pseudo-labels (indicating the categories to which the samples belong) and negative pseudo-labels (indicating the categories to which the samples do not belong). In the third stage, the data with pseudo-labels and a small amount of labeled data are both input into the network for supervised training. Then the second and third stages are performed iteratively until the preset conditions are reached.

In the above self-training process, the proposed SGSL model can learn a graph structure between labeled and unlabeled samples in the third stage, which is conducive to promoting the features of unlabeled data to gradually become closer to those of labeled data, ensuring that the predictions of unlabeled data are consistent with those of labeled data. In addition, the proposed UGCN includes a dropout-based graph convolutional network and an uncertainty filtering process. During the first and third stages, the dropout-based graph convolutional network can aggregate neighborhood features based on the learned graph structures, making similar features more similar in the data space. Moreover, in the second stage, the UGCN outputs both predictions and uncertainties, and judges the credibility of predictions through a double verification strategy. This results in the generation of pseudo-labels with less noise. This is beneficial for network training and further improves the performance of self-training.

The proposed method improves the quality of generated features by considering the similarity between samples and reduces the noise of pseudo-labels based on uncertainty. Our approach is well-adapted for tasks that require measuring similarity between samples, such as clustering and retrieval tasks. Therefore, in this paper, image clustering and person re-identification are chosen as case problems to evaluate the performance of the proposed method. In these two tasks, the input data can be modeled as graph structures so that the proposed semi-supervised approach can be applied.

The contributions of this paper are as follows:

(1) A SGSL model is proposed to consider the potential correlation between labeled data and unlabeled data. It calculates the similarity between unlabeled and labeled sample features in a batch to initialize their correlation. Moreover, end-to-end training makes this correlation optimized, which facilitates the network to learn more discriminative features and, thus, makes the confidence of predictions more accurate and credible.

(2) In order to improve the accuracy and reliability of pseudo-labels, the UGCN is proposed. It uses the graph convolutional network to aggregate features based on the learned graph structures so that the unlabeled sample features are close to the similar labeled sample features. When features are passed through the network, the predictions will be consistent and, thus, improve the prediction accuracy of unlabeled samples. In addition, we also use dropout to obtain the uncertainty of predictions. If the uncertainty of predictions is high, it means that the confidence is not credible and does not generate pseudo-labels for the corresponding samples to improve the reliability of pseudo-labels.

(3) A positive and negative self-training framework based on graph-based deep uncertainty is proposed, which fuses the proposed SGSL and UGCN in the self-training framework. It can make features more discriminative in data space and improve the accuracy of pseudo-labels when the framework is trained end-to-end.

## 2. Related Work

Semi-supervised learning methods can be broadly divided into two categories: consistency regularization methods [1,2] and pseudo-labeling methods [3,4]. There are three kinds of perturbations in consistency regularization methods, i.e., perturbations to inputs [1,9], perturbations to the network [10], and perturbations to the training process [2,11]. Applying perturbations to inputs is the most used strategy. For example, Guyon et al. [12] propose the mean teacher model, which consists of two parts: the student model and teacher model. Images are augmented twice and then inputted into the student model and the teacher model to predict the corresponding label distributions, respectively, after which a consistency loss is utilized for both predictions. Ke et al. [13] propose the dual student method, which replaces the teacher model in the mean teacher method. When applying perturbations to the network, Zhang et al. [10] propose the worst-case perturbation method, in which additive and DropConnect perturbation are used to the network. Methods using perturbations to inputs are widely studied. However, these methods rely on data augmentation strategies. Their performances will be limited if consistent regularization methods are utilized in areas where the effectiveness of data augmentation is low (e.g., video, medical images).

Pseudo-labeling methods generate pseudo-labels for unlabeled data and then train the network. Pseudo-labeling methods can be divided into two categories, i.e., multi-view training methods [3,4,14,15] and self-training methods [6,7].

Multi-view training methods focus on training two or more different networks and providing pseudo-labels to each other. For instance, the co-training method [3] contains two networks that take images from two views as inputs. If one of the networks has higher confidence, then pseudo-labels will be generated for inputs and served as the training set for the other network in the next iteration. Chen et al. [14] propose a method with three networks. If the predictions of the two models are consistent, then pseudo-labels are further generated, which are then used as the training data for the third model in the next iteration. In multi-view training methods, it is inevitable that multiple networks are involved and, thus, the volume of network parameters to be trained increases, making it difficult to apply to scenarios with limited resources.

In contrast, self-training methods use a single network to predict and generate pseudo-labels. For example, Lee et al. [6] propose pseudo-labeling methods in which the network was trained using a supervised learning approach via a small amount of labeled data; the trained network model is used to predict unlabeled data. The predictions are filtered to generate pseudo-labels and are added to the training set to train the network iteratively. Xie et al. [16] propose a noisy student model, which consists of a teacher model and a student model. The teacher model is firstly trained on a small amount of labeled data, and then the teacher model is used to predict the unlabeled data and generate pseudo-labels. The pseudo-labeled data and the labeled data are then combined and trained with the student model, which becomes a new teacher model after training and is trained again iteratively by re-predicting the unlabeled data. Self-training methods do not rely on data augmentation strategies and their network parameters are greatly reduced compared to multi-view training methods. The main drawback of these methods is that the generated pseudo-labels are not always accurate. To reduce the noise in pseudo-labels, Rizve et al. [8] propose using uncertainty to determine whether predictions are reliable. Moreover, the higher the uncertainty, the less reliable the predictions. In addition, Rizve et al. [8] argue that the predictions with low confidence can also be used to generate pseudo-labels to perform negative learning. However, this method does not take into account the correlation between labeled data and unlabeled data during network training.

There are also many graph-based semi-supervised learning methods [17,18], in which all data are represented as nodes in a graph, and labels of unlabeled data are obtained by label propagation. These methods generally carry out research in terms of both graph construction [19] and label inference [20]. Unlike them, graphs are used in our approach to model the deep similarity between samples, which can be used for graph convolution to optimize the feature distribution and, thus, improve the quality of the generated pseudo-labels.

## 3. Methods

### 3.1. Overview

**Figure 1:**
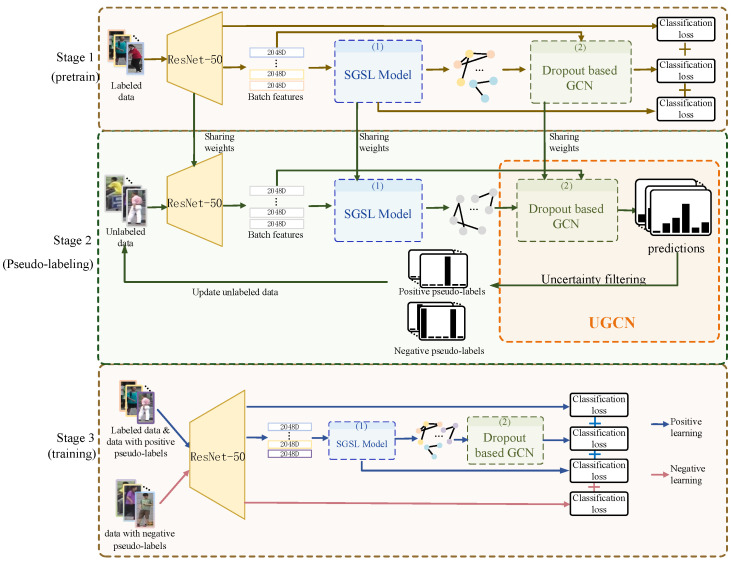
A positive and negative self-training framework based on graph-based deep uncertainty. The proposed framework is divided into three stages. In the first stage, a small number of labeled samples are fed into the network and trained using a supervised learning approach. In the second stage, the parameters are fixed and the network is tuned to the test mode. Unlabeled samples are inputted to predict and generate pseudo-labels by uncertainty filtering. Then original unlabeled data are updated by adding positive and negative pseudo-labels. In the third stage, the data with pseudo-labels are trained together with a small amount of labeled data.

To learn the correlation between labeled and unlabeled data, we propose a positive and negative self-training framework based on graph-based deep uncertainty, as shown in Figure 1.

Given an image training set $I=\{I^{1},I^{2},\ldots ,I^{O}\}$, where *O* represents the number of images in I. In semi-supervised settings, the training set is divided into two sets, i.e., labeled images $I_{L}=\left\{I^{1},I^{2},\ldots ,I^{L}\right\}$ and unlabeled images $I_{U}=\left\{I^{1},I^{2},\ldots ,I^{U}\right\}$, where *L* is the number of labeled images and *U* is the number of unlabeled images, L≪U, $I=I_{L}\cup I_{U}$. The proposed positive and negative self-training framework based on graph-based deep uncertainty has three stages, which can be described as follows.

In the first stage, $I_{L}$ is passed through ResNet-50 in batches to obtain the batch features $X_{b}^{L}$ and $X_{b}^{L}\in R^{b\times dim}$, where *b* denotes the number of images in a batch and dim is the dimension of features. The batch features are directly used to generate predictions. After that, the predictions of batch features logits and labels are inputted into the classification loss, where $logits\in R^{b\times M}$ and *M* is the number of classes. Batch features $X_{b}^{L}$ are also inputted to the proposed SGSL model, which outputs the correlation $A_{b}^{L}\in R^{b\times b}$ between samples in the current batch. Then $A_{b}^{L}$ and the true correlation between samples in the current batch, denoted as $A^{tar}$, are inputted to binary classification loss. Moreover, $A_{b}^{L}$ and $X_{b}^{L}$ are inputted to the proposed dropout-based GCN, which outputs predictions $logits_{g}nn\in R^{b\times M}$. Then $logits_{g}nn$ and labels are inputted to classification loss.

In summary, the losses in this phase consist of three items: (a) the loss between the generated similarity graph of SGSL and the true relationship graph between samples, which supervise the training of ResNet-50 and SGSL; (b) the loss between the predictions of UGCN and the ground truth labels of samples, which supervise the training of ResNet-50, SGSL, and UGCN; (c) the loss generated directly from the classification from the batch features; this loss supervises the training of ResNet-50.

In the second stage, the trained network is used to extract features of $I_{U}$ in batches, i.e., $X_{b}^{U}$ and $X_{b}^{U}\in R^{b\times dim}$. Then $X_{b}^{U}$ is input to the SGSL model to obtain the correlation $A_{b}^{U}\in R^{b\times b}$ of features in that batch of data. After that, $X_{b}^{U}$ and $A_{b}^{U}$ are input to the proposed UGCN to generate positive and negative pseudo-labels for unlabeled data. In this stage, the weights of the model are fixed.

In the third stage, the network is trained based on the pseudo-labeled samples obtained in the second stage together with the original samples with labels. The positive and negative self-training is performed in this phase. The training process of positive learning is the same as the first stage. Moreover, for negative learning, $I_{U}$ is fed into ResNet-50, then the predictions $logits_{neg}\in R^{b\times M}$ are output. $logits_{neg}$ and negative pseudo-labels are inputted to negative cross-entropy loss. More specifically, after obtaining pseudo-labels for the unlabeled data, where positive pseudo-labels represent the categories to which the samples belong and negative pseudo-labels indicate the categories to which the samples do not belong, both positive and negative labels are used as inputs to the cross-entropy loss function to supervise the model to learn features with discriminative properties. The difference is that for positive pseudo-labels, the model predicts the category the sample belongs to, while for negative pseudo-labels, the model predicts the category the sample does not belong to. In addition, the usage of the original ground truth labels is the same as the positive pseudo-labels.

In the self-training process, the second and third stages are iterated until the number of iterations reaches the preset number $NUM_{iter}$.

### 3.2. The Similarity Graph Structural Learning Model

**Figure 2:**
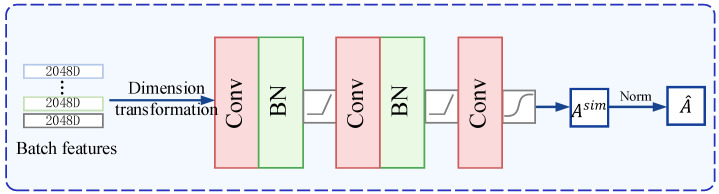
The architecture of the SGSL model. Input batch sample features $X_{b}$. After dimension transformation, the initial representations of structural correlations can be obtained. Then after the convolutional layer, batch normalization, and activation function, the correlation between batch sample features is learned.

In order to take into account the correlation between labeled and unlabeled samples in semi-supervised learning, so that the unlabeled sample features can be close to their corresponding labeled sample features, and to make the predictions of unlabeled samples more credible, we propose a SGSL model to learn the correlation between labeled and unlabeled samples, as shown in Figure 2.

Given batch features $X_{b}\in R^{b\times dim}$, the purpose of the proposed SGSL model is to learn the similarity graph structure $\hat{A}\in R^{b\times b}$. At first, the dimension of batch features $X_{b}$ is transformed by adding a dimension, i.e., $X_{b}\in R^{1\times b\times dim}$. Then, we swap the first and second dimensions of $X_{b}$ to obtain $X_{b}^{{}^{\prime }}$ and $X_{b}^{{}^{\prime }}\in R^{b\times 1\times dim}$. Next, $X_{b}$ and $X_{b}^{{}^{\prime }}$ are subtracted to obtain the initialized representations $A^{fea}$ of the similarity graph structure, i.e., $A^{fea}=X_{b}-X_{b}^{{}^{\prime }}$ and $A^{fea}\in R^{b\times b\times dim}$. The entry of *i*-th row and *j*-th column of $A^{fea}$ denote the correlation representation of the *i*-th sample and *j*-th sample in the batch and it has a dimension dim. Then, $A^{fea}$ is fed into the proposed SGSL model, which consists of convolutional layers, batch normalization, and activation functions. Each convolutional layer has a kernel size of 1×1 and a stride of 1×1. The input dimension of the first convolutional layer is dim and the output dimension is $dim_{conv1}^{out}$, the input dimension of the second convolutional layer is $dim_{conv1}^{out}$, and the output dimension is $dim_{conv2}^{out}$. After the second convolutional layer, the input dimension of the third convolutional layer is $dim_{conv2}^{out}$ while the output dimension is 1 because the similarity graph structure of the batch samples needs to be obtained. After the sigmoid function, the structure $A^{sim}$ between the batch samples is obtained, the values in $A^{sim}$ are all between 0 and 1, and $A^{sim}\in R^{b\times b}$. Then, $A^{sim}$ is normalized, i.e.,

(1) $$\hat{A}=D^{-\frac{1}{2}}\left(A^{sim}+J\right)D^{-\frac{1}{2}}$$

where *D* is the diagonalized degree matrix and J represents the identity matrix.

During the training process, the graph structure $A^{tar}$ of the current batch of samples is obtained based on their true labels or pseudo-labels, as specified by the following rules

$$A_{i,j}^{tar}=\left\{\begin{array}{cc} 1 & ,\;x^{i}\;\mathrm{and}\;x^{j}\;\mathrm{have}\;\mathrm{the}\;\mathrm{same}\;\mathrm{label}\;\mathrm{or}\;\mathrm{pseudo-label}\\ 0 & ,\;\mathrm{otherwise} \end{array}\right.$$

Then, $A^{tar}$ and $A^{sim}$ are input to a binary cross-entropy loss, i.e.,

$$L_{SR}=A_{i,j}^{tar}\log\left(A_{i,j}^{sim}\right)+\left(1-A_{i,j}^{tar}\right)\log\left(1-A_{i,j}^{sim}\right)$$

Moreover, the data input into SGSL to model similarity differ in the three phases. In stage 1, SGSL is in training mode, and all the input data are labeled data with real labels; in stage 2, the weights of SGSL are fixed, and the similarity between the input data (including labeled data and unlabeled data) is evaluated; in stage 3, SGSL is in a training mode, the input data consist of labeled data and unlabeled data with positive pseudo-label, and the labels consist of real labels and positive pseudo-labels.

### 3.3. Uncertainty-Based Graph Convolutional Network

**Figure 3:**
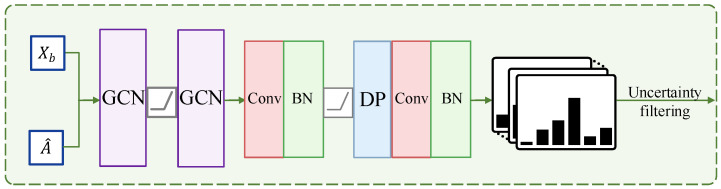
The architecture of the proposed UGCN. Similarity graph structure $\hat{A}$ and batch features $X_{b}$ are input to UGCN. After the two-layer GCN, features with neighbor aggregation $F^{\left(3\right)}$ are obtained. Then, $F^{\left(3\right)}$ and $X_{b}$ are concatenated and input to the classifier. “Conv” represents the convolutional layer, “BN” is batch normalization, and “DP” denotes dropout.

In order to make the features of unlabeled data close to the features of corresponding labeled data, so that similar features are consistent in prediction, and to use uncertainty to determine whether the prediction confidence is reliable, UGCN is proposed, as shown in Figure 3.

Given batch features $X_{b}$ and the output of SGSL model $\hat{A}$, UGCN firstly uses the graph convolution network to aggregate features based on the similarity graph structure $\hat{A}$, i.e.,

(2) $$F^{\left(l+1\right)}=\sigma \left(\hat{A}⊙F^{\left(l\right)}⊙\theta_{gcn}^{\left(l\right)}\right)$$

where $F^{\left(l\right)}$ is the input of *l*-th GCN and $F^{\left(1\right)}=X_{b}$. ⊙ denotes the inner product. $\theta_{gcn}^{\left(l\right)}$ represents the learnable parameter of *l*-th GCN, and $\theta_{gcn}^{\left(l\right)}\in R^{dim_{in}^{\left(l\right)}\times dim_{out}^{\left(l\right)}}$. σ is the activation function. After GCNs, the aggregated features $F^{\left(3\right)}\in R^{b\times dim_{out}^{\left(2\right)}}$ can be obtained, i.e.,

(3) $$F^{\left(3\right)}=\hat{A}⊙\left(\sigma \left(\hat{A}⊙F^{\left(1\right)}⊙\theta_{gcn}^{\left(1\right)}\right)\right)⊙\theta_{gcn}^{\left(2\right)}$$

Then, $F^{\left(3\right)}$ and $X_{b}$ are concatenated, i.e.,

(4) $$X_{b}^{agg}=concat\left(X_{b},F^{\left(3\right)}\right)$$

where concat represents concatenation along the feature dimension, $X_{b}^{agg}\in R^{b\times (dim+dim_{out}^{\left(2\right)})}$. Then $X_{b}^{agg}$ is input to the convolutional layer. After batch normalization, activation function, and dropout, a convolutional layer and batch normalization are attached to obtain the predictions of $X_{b}^{agg}$, i.e., ${\hat{Y}}_{b}$, ${\hat{Y}}_{b}\in R^{b\times M}$. The output dimension of the second convolutional layer is *M*.

The above process is the training process in the first and third stages. While in the second stage, UGCN is able to output the uncertainty of predictions for generating pseudo-labels. The uncertainty is obtained by dropout. Specifically, the model is in the test mode in the second stage, but the dropout layer is in the training mode. Therefore, the predictions are different when inputting the same samples twice. The standard deviation can be used to measure whether the predictions are credible. he proposed method repeatedly inputs each sample in a batch *T* times to obtain *T* predictions. Then a sigmoid function is used to restrict the values between 0 and 1. After that, the average of the results obtained from the *T* predictions is calculated, i.e.,

(5) $${\hat{Y}}_{b}=\frac{1}{T}\sum_{t=1}^{T}{\hat{Y}}_{b}^{\left(t\right)}$$

where ${\hat{Y}}_{b}^{\left(t\right)}$ represents the output of *t*-th inputs, *T* denotes the number of times that data are repeatedly fed into the network, T=10 in the proposed method. ${\hat{Y}}_{b}$ denotes the predictions in the second stage and ${\hat{Y}}_{b}\in R^{b\times M}$. Then the maximum value in ${\hat{Y}}_{b}$ can be obtained,

(6) $${\hat{P}}_{b}=max\left({\hat{Y}}_{b}\right)$$

where ${\hat{P}}_{b}$ represents the confidence of samples belonging to the corresponding class, ${\hat{P}}_{b}\in R^{b\times 1}$. For uncertainty, the standard deviation is calculated,

(7) $$U_{b}=std\left({\hat{Y}}_{b}^{\left(1\to T\right)}\right)$$

where ${\hat{Y}}_{b}^{\left(1\to T\right)}$ is *T* times the outputs of the same batch samples, and ${\hat{Y}}_{b}^{\left(1\to T\right)}\in R^{T\times b\times M}$, std calculates the standard deviation across the first dimension, and $U_{b}\in R^{b\times M}$. Next, the standard deviation ${\hat{U}}_{b}$ corresponding to the maximum predicted value in ${\hat{P}}_{b}$ is obtained and ${\hat{U}}_{b}\in R^{b\times 1}$. Finally, the prediction confidence ${\hat{P}}_{b}$ of a batch sample and its corresponding uncertainty ${\hat{U}}_{b}$ are obtained.

In summary, the role and training of UGCN in three phases are as follows: (a) In the first stage, UGCN is set as the training mode, and the sample features extracted by ResNet-50 are aggregated in the neighborhood according to the similarity graph built by SGSL, the predicted categories of the samples are output after graph convolution. In this process, because the inputs are labeled data, ground truth labels supervise the training of UGCN. (b) In the second stage, UGCN is set as the eval mode. The inputs to the network are unlabeled data, and UGCN predicts these samples to obtain their pseudo-labels. Moreover, the UGCN generates confidence for the prediction of each sample as an assist to the pseudo-label generation. In this process, the weights of UGCN a fixed. (c) In the third stage, the UGCN is set to the training mode. The input of the network consists of labeled data and unlabeled data with pseudo-labels, and the UGCN performs graph convolution on the similarity graph of these data to output predictions, ground truth labels, and pseudo-labels, generating losses to supervise its training.

### 3.4. Pseudo-Label Generation Based on Uncertainty

We utilize a pseudo-label generation method based on uncertainty. Given prediction confidence ${\hat{P}}_{b}$ and corresponding uncertainty ${\hat{U}}_{b}$, the *i*-th sample in the batch has a positive pseudo-label only if the following condition is satisfied,

(8) $${\hat{U}}_{b}\left(i\right)\leq \kappa_{p}\wedge {\hat{P}}_{b}\left(i\right)\geq \tau_{p}$$

where ${\hat{U}}_{b}\left(i\right)$ is the prediction confidence of the *i*-th sample in the batch and ${\hat{P}}_{b}\left(i\right)$ is the corresponding uncertainty. $\kappa_{p}$ and $\tau_{p}$ are predefined values used to filter the uncertainty and prediction confidence, respectively. If the prediction confidence of sample $x^{i}$ is greater than or equal to $\tau_{p}$, and its uncertainty is less than $\kappa_{p}$, then the prediction confidence is considered reliable, and a positive pseudo-label can be generated. Such a strategy leaves many unlabeled samples unlabeled, but in fact, although these samples do not obtain positive pseudo-labels, they can obtain negative pseudo-labels, i.e., to determine the categories to which these samples explicitly do not belong to, the specific rule is,

(9) $${\hat{U}}_{b}\left(j\right)\leq \kappa_{n}\wedge {\hat{P}}_{b}\left(j\right)\leq \tau_{n}$$

where $\kappa_{n}$ and $\tau_{n}$ are pre-defined values used to filter the uncertainty and prediction confidence for negative pseudo-labels. If the sample $x^{j}$ fails to be assigned to a positive pseudo-label, a prediction confidence less than $\tau_{n}$, and an uncertainty value less than $\kappa_{n}$, then it can be considered that $x^{j}$ does not belong to the class corresponding to that prediction confidence and the corresponding position. After this process, the generated positive and negative pseudo-labels are used to update the original unlabeled data, and then in the third stage, the positive and negative pseudo-labeled data are used to train the network together with the original labeled data.

## 4. Results and Discussion

### 4.1. Datasets and Settings

Our approach is suitable for tasks that are sensitive to inter-sample connections, such as clustering and retrieval tasks. The proposed method is evaluated on image clustering and person re-identification (re-ID) tasks. In these two tasks, the data can be naturally modeled as graph structures, which allows learning the similarity between samples. Since the inputs are image data, the general and powerful CNN model ResNet-50 [21] is used as the feature extractor. Our semi-supervised approach improves the performance of the model by increasing the accuracy of pseudo-labels. To evaluate the proposed method, we adopt the metrics used in previous works.

For image clustering tasks, IJB-B [22] and IJB-C [23] datasets are utilized. In the IJB-B dataset, there are seven subsets for clustering. In this paper, the top 3 subsets with the most images are selected for clustering, i.e., the subsets including 512, 1024, and 1845 identities. Moreover, in these subsets, there are 18,251, 36,575, and 68,195 images, respectively. The IJB-C dataset is an upgraded version of the IJB-B dataset, which has 4 subsets with 32, 1021, 1839, and 3531 identities, respectively. The top 3 subsets with the largest image numbers are also selected for clustering, and these subsets include 41,074, 71,392, and 140,623 images, respectively. The widely used normalized mutual information (NMI) is our evaluation metric for image clustering. In semi-supervised settings, only one-third of images of each subset are labeled, the rest of the labels are not involved in semi-supervised training.

For the person re-ID task, Market-1501 [24] and DukeMTMC-reID [25] datasets are used. Market-1501 includes 32,668 images of 1501 pedestrians captured by 6 cameras from different angles. There are 12,936 images from 751 pedestrians in the training set, 19,732 images from another 750 pedestrians in the gallery set, and 3368 images in the query set. The DukeMTMC-reID dataset contains 36,411 images of 1401 pedestrians and is captured by 8 cameras from different angles. There are 16,522 images of 702 pedestrians in the training set, 17,661 images of another 702 pedestrians in the gallery set, and 2228 images in the query set. For evaluation, the widely used mean average precision (mAP) and cumulative match characteristic (CMC) curve are calculated. For semi-supervised learning, only 1/3 of the labels in the training set are available, the rest of the labels are not involved in semi-supervised training.

### 4.2. Implementation Details

The proposed method was implemented using the PyTorch deep learning framework, including torch 1.10.0, cudnn 8.2.0, and CUDA 11.3. The Python version used was 3.8.5. The server hardware consisted of an NVIDIA Geforce RTX 3090 and an Intel(R) Core(TM) i9-10900K CPU @ 3.70 GHz. The operating system used was Ubuntu 20.04.3 LTS.

The original images were all resized to 256×128 and randomly horizontally flipped for data augmentation. The stochastic gradient descent (SGD) algorithm was utilized to optimize the proposed model with an initial learning rate of 0.03; the momentum is 0.9. Here, $NUM_{iter}=20$, and in each iteration, the proposed model was trained for 60 epochs. In addition, $\tau_{p}=0.8$, $\tau_{n}=0.05$, $\kappa_{p}=0.05$, and $\kappa_{n}=0.005$.

### 4.3. Ablation Study

To explore the impact of the proposed SGSL model and UGCN, ablation experiments were conducted on the Market-1501 dataset, as shown in Table 1.

In Table 1, “*w*/*o* UGCN” indicates that the SGSL model and UGCN are removed from the proposed method, “*w*/*o* Uncertainty” indicates that uncertainty is not utilized in generating pseudo-labels, and “Proposed” indicates the proposed method.

As shown in Table 1, compared to variant 1, variant 3 improves mAP by 3.8%, Rank-1 by 2.8%, Rank-5 by 1.5%, Rank-10 by 1.2%, and Rank-20 by 0.8%. The difference between variant 3 and variant 1 is that variant 3 utilizes the proposed SGSL model and UGCN, and the experimental results are improved because the SGSL model considers the correlation between unlabeled and labeled samples. This correlation is then input to the graph convolutional network. With the feature aggregation capability of UGCN, it can make the features of the unlabeled samples approach its similarly labeled samples gradually, and then drive the unlabeled samples to obtain more reliable classification predictions.

In addition, variant 3 improved mAP by 4.2%, Rank-1 by 3.2%, Rank-5 by 2.0%, Rank-10 by 1.4%, and Rank-20 by 0.9% compared to variant 2. The main difference between the two sets of experiments is that in variant 3, the proposed method utilizes uncertainty to assist in generating pseudo-labels for unlabeled samples. The main reason for the improved results is that the pseudo-label generation for unlabeled samples in variant 2 relies entirely on predictions of the network. However, if there are incorrect predictions, the generated pseudo-labels are more likely to be noisy and lead the network to be trained in the wrong direction. In variant 3, the same batch of samples is repeatedly fed into the network 10 times and the standard deviation of the prediction results is calculated. This standard deviation is used as the uncertainty of predictions. Then, pseudo-labels are generated by filtering the predictions with low uncertainty, which effectively reduces the noise in pseudo-labels and leads to an improvement in the network’s performance. Therefore, the performance of variant 3 is better than that of variant 2.

### 4.4. Parameters Analysis

In the following experiments, the influence of threshold $\tau_{p}$ and GCN layer *l* on the performance is explored.

**Figure 4:**
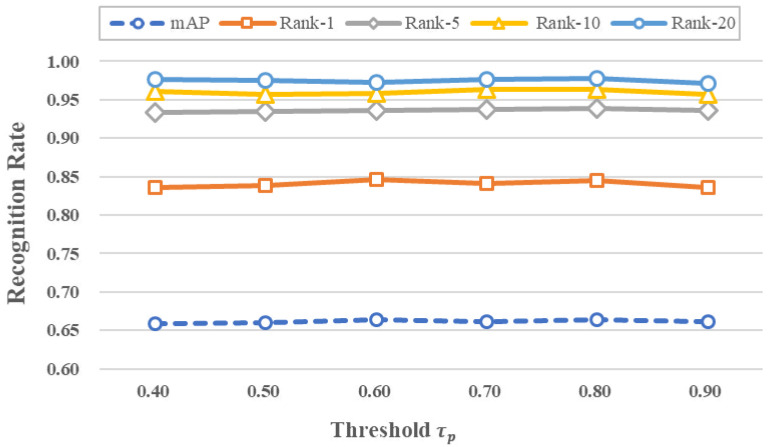
The influence of $\tau_{p}$ on performance.

To explore the impact of the threshold value $\tau_{p}$, we varied it from 0.4 to 0.9 in increments of 0.1, with the number of GCN layers set to 2. The experimental results are presented in Figure 4, and specific numerical results are provided in Table 2. Figure 4 shows that the performance of the model is relatively stable on mAP, Rank-1, Rank-5, Rank-10, and Rank-20 with varying values of $\tau_{p}$, and most of the evaluation metrics achieve their best results when $\tau_{p}$ is set to 0.8. This part of the results shows that $\tau_{p}$ brings less influence to the proposed method. The possible reason is that the features of the model tend to be distinguishable and stable after several iterations of the self-training process when the pseudo-labels predicted by UGCN tend to be correct and have a high confidence level. Therefore adjusting the confidence threshold does not affect the model to select the true positive samples.

**Figure 5:**
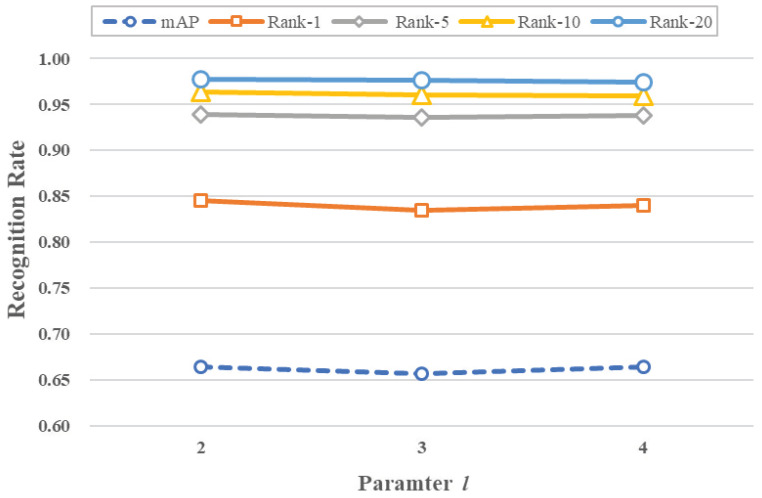
The influence of *l* on performance.

In the experiments exploring the effect of the number of graph convolution layers *l*, we set *l* to 2, 3, and 4, respectively, with $\tau_{p}$ set to 0.8. The experimental results are shown in Figure 5, and the specific numerical results are shown in Table 3. It can be observed from Figure 5 that the model’s performance remains relatively stable as the number of graph convolution layers changes, with most of the tested metrics reaching their best performance when the model has two layers of graph convolution. This part of the experimental results shows that the model is less sensitive to the number of graph convolution layers. This is likely because increasing the depth of the graph convolution introduces an additional number of parameters. In semi-supervised training, most of the data are unlabeled data. Increasing the depth of the network does not effectively increase the knowledge gained by the model from the data, so changing the number of layers of the graph convolution has little effect on the performance of the network and may even bring about a decrease in performance.

### 4.5. Runtime Analysis

The running time of the model in each phase of the proposed method is shown in Table 4. The results in the table are measured with a batch size of 64. From Table 4, it can be seen that stage 2 of generating pseudo-labels and performing uncertainty filtering takes the longest time in training. That is probably because it has to traverse and filter the confidence of samples to obtain positive and negative pseudo-labels. In the testing phase, the model is able to process about 1800 images per second, thus providing a certain level of the real-time performance.

### 4.6. Performance Comparison

#### 4.6.1. Comparison of the Image Clustering Task

**Figure 6:**
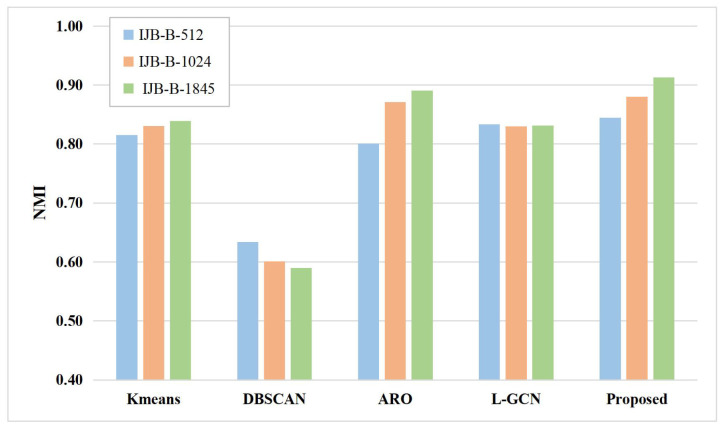
Performance comparison on the IJB-B dataset.

**Figure 7:**
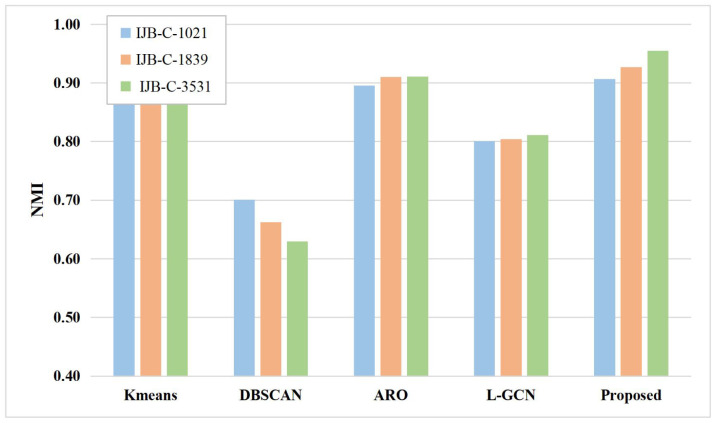
Performance comparison on the IJB-C dataset.

The proposed method is compared to the classical clustering methods. For a fair comparison, the features extracted by the proposed method are used for the rest of the clustering methods. The experimental results are shown in Figure 6 and Figure 7. The specific numerical results are shown in Table 5 and Table 6.

As shown in Figure 6 and Figure 7, the proposed method outperforms the remaining clustering methods in terms of experimental results. For example, on the IJB-B-512 subset, the proposed method improves by 2.89% compared to k-means, 21.07% compared to the DBSCAN method, 4.4% compared to the ARO method, and 1.14% compared to the L-GCN, and achieves similar results to the rest of the IJB-B subsets. The experimental results show that the predictions of the proposed method have high accuracy. This is mainly because, the proposed method improves the accuracy of predictions from two perspectives, i.e., the discrimination of features and the accuracy of pseudo-labeling. Specifically, the proposed method learns the similarity graph structure between labeled and unlabeled samples using the SGSL model, and then makes the features more discriminative by UGCN. Moreover, when generating pseudo-labels for unlabeled samples, the proposed method not only uses uncertainty to check the reliability of prediction confidence, but also makes full use of samples with low confidence and generates negative pseudo-labels for them to enrich supervised information of the network.

#### 4.6.2. Comparison of Person Re-ID Task

**Figure 8:**
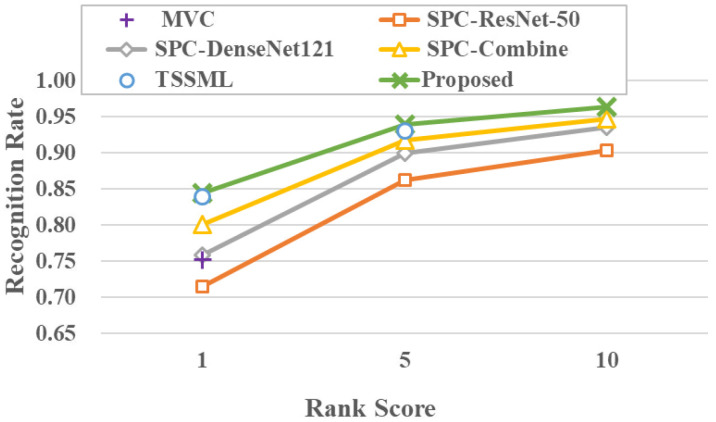
Performance comparison on the Market-1501 dataset.

**Figure 9:**
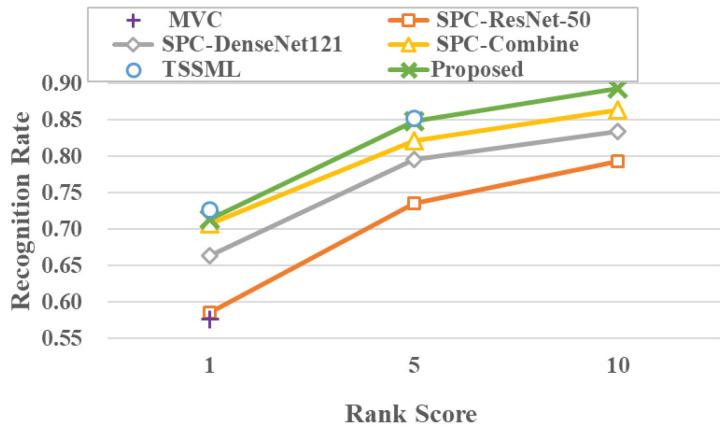
Performance comparison on the DukeMTMC-reID dataset.

The proposed method is being compared to semi-supervised person re-identification methods. For a fair comparison, the proposed method is only compared to those methods with the same semi-supervised setup. These methods can be briefly described as follows: MVC [30], which is a semi-supervised method based on self-training, SPC [31], which is a semi-supervised method based on self-paced learning, and TSSML [32], which is a person re-identification method based on transductive learning. The experimental results on the Market-1501 and DukeMTMC-reID datasets are shown in Figure 8 and Figure 9, and the specific numerical results are shown in Table 7 and Table 8, respectively.

From the comparison results on the Market-1501 dataset, it can be seen that the proposed method achieves the best results on mAP, Rank-1, Rank-5, and Rank-10. Compared to the suboptimal TSSML method, the proposed method improves by 0.8% on mAP, 0.6% on Rank-1, and 0.8% on Rank-5. Moreover, from the comparison results on the DukeMTMC-reID dataset, it shows that the proposed method improves by 0.6% in mAP compared to the TSSML method, while it is still competitive in Rank-1 and Rank-5, although it is not the best. Compared to the SPC-Combine method, the proposed method improves by 3.6% on mAP, 0.7% on Rank-1, 2.7% on Rank-5, and 2.9% on Rank-10.

There are two main reasons for the strong competitiveness of the proposed method. Firstly, we fully consider the potential correlation between labeled and unlabeled samples during training. Then, we exploit the neighborhood aggregation capability of the graph convolutional network to gradually drive the features of unlabeled samples to approach those of similar labeled samples during training. This, in turn, drives the backbone network to learn more discriminative features through backpropagation. Secondly, to reduce the noise in pseudo-labels, uncertainty is utilized to measure the reliability of predictions by repeatedly feeding batch samples into the network 10 times and calculating the standard deviation of 10 results. Only those with a standard deviation less than a threshold are considered reliable classification predictions. Therefore, the experimental results of the proposed method on both image clustering and person re-identification tasks are highly competitive, demonstrating that the proposed method can learn more discriminative features and generate more accurate pseudo-labels.

## 5. Conclusions

We propose a positive and negative self-training framework based on graph-based deep uncertainty, which can utilize the potential correlation between labeled and unlabeled samples in semi-supervised learning. The network includes two key models, i.e., SGSL and UGCN. The SGSL model builds a kind of similarity graph structure for labeled and unlabeled samples. The UGCN can aggregate features in the training phase based on the learned graph structure, making the features more discriminative. In addition, it can output uncertainty for predictions in the pseudo-label generation phase and generate pseudo-labels only for the unlabeled samples with low uncertainty, which in turn reduces the noise in pseudo-labels. The proposed method is evaluated on image clustering and person re-identification tasks, and both experimental results show the effectiveness of the proposed method.

## Figures and Tables

**Table 1:** Ablation study on the Market-1501 dataset.

Variants	Model Setting	mAP	Rank-1	Rank-5	Rank-10	Rank-20
1	*w*/*o* UGCN	62.6	81.7	92.4	95.2	97.0
2	*w*/*o* Uncertainty	62.2	81.3	91.9	95.0	96.9
3	Proposed	**66.4**	**84.5**	**93.9**	**96.4**	**97.8**

**Table 2:** The influence of $\tau_{p}$ on performance.

$\tau_{p}$	mAP	Rank-1	Rank-5	Rank-10	Rank-20
0.40	65.9	83.6	93.4	96.1	97.6
0.50	66.0	83.9	93.5	95.7	97.5
0.60	66.4	84.6	93.6	95.8	97.3
0.70	66.1	84.1	93.8	96.4	97.7
0.80	66.4	84.5	93.9	96.4	97.8
0.90	66.2	83.6	93.6	95.7	97.1

**Table 3:** Parameter *l* influence on the performance.

*l*	mAP	Rank-1	Rank-5	Rank-10	Rank-20
2	66.4	84.5	93.9	96.4	97.8
3	65.7	83.5	93.6	96.1	97.6
4	66.4	84.0	93.8	96.0	97.4

**Table 4:** Running time of the model on a batch in each phase.

Phase		Runtime
	Stage 1	220 ms
Training	Stage 2	365 ms
	Stage 3	160 ms
Testing		35 ms

**Table 5:** Method comparison on the three subsets of IJB-B.

Methods	NMI		
	IJB-B-512	IJB-B-1024	IJB-B-1845
K-Means [26]	0.8149	0.8303	0.8393
DBSCAN [27]	0.6340	0.6007	0.5896
ARO [28]	0.8007	0.8714	0.8904
L-GCN [29]	0.8333	0.8301	0.8311
Proposed	**0.8447**	**0.8802**	**0.9128**

**Table 6:** Method comparison on the three subsets of IJB-C.

Methods	NMI		
	IJB-C-1021	IJB-C-1839	IJB-C-3531
K-Means [26]	0.8690	0.8674	0.8676
DBSCAN [27]	0.7010	0.6625	0.6297
ARO [28]	0.8955	0.9101	0.9111
L-GCN [29]	0.8008	0.8042	0.8111
Proposed	**0.9063**	**0.9271**	**0.9548**

**Table 7:** Performance comparison on the Market-1501 dataset.

	mAP	Rank-1	Rank-5	Rank-10
MVC [30]	52.6	75.2	-	-
SPC-ResNet50 [31]	53.2	75.1	86.2	90.3
SPC-DenseNet101 [31]	56.6	75.9	90.0	93.5
SPC-Combine [31]	62.8	80.1	91.8	94.7
TSSML [32]	65.6	83.9	93.1	-
proposed	**66.4**	**84.5**	**93.9**	**96.4**

**Table 8:** Performance comparison on the DukeMTMC-reID dataset.

	mAP	Rank-1	Rank-5	Rank-10
MVC [30]	37.8	57.6	-	-
SPC-ResNet50 [31]	37.4	58.5	73.6	79.3
SPC-DenseNet101 [31]	45.1	66.3	79.6	83.4
SPC-Combine [31]	50.2	70.7	82.1	86.4
TSSML [32]	53.2	**72.7**	**85.2**	-
proposed	**53.8**	71.4	84.8	**89.3**

## Data Availability

Four publicly available datasets (IJB-B, IJB-C, Market-1501, and DukeMTMC-reID) were used to illustrate and evaluate the proposed method.

## Abbreviations

GCN: graph convolution neural network.
